# Does Parathyroidectomy Affect the Neutrophil/Lymphocyte Ratio, a Systemic Inflammatory Marker?

**DOI:** 10.7759/cureus.13708

**Published:** 2021-03-05

**Authors:** Hüseyin Alakuş, Mustafa Göksu

**Affiliations:** 1 Department of Surgical Oncology, Adiyaman University Faculty of Medicine, Adiyaman, TUR; 2 Department of General Surgery, Adiyaman University Faculty of Medicine, Adiyaman, TUR

**Keywords:** primary hyperparathyroidism, neutrophil-to-lymphocyte ratio, neutrophils, lymphocytes, parathyroidectomy

## Abstract

Introduction

Primary hyperparathyroidism (PHPT) is an endocrinological disorder associated with increased systemic inflammation. This study aimed to examine the changes in the neutrophil/lymphocyte ratio (NLR), serum parathormone, serum corrected calcium, serum phosphate, and white blood cell (WBC) count ​​in patients with PHPT before and after parathyroidectomy.

Methods

A total of 37 patients who underwent successful parathyroidectomy for PHPT were included in the study. NLR, serum parathormone, serum corrected calcium, serum phosphate, and WBC count ​​were compared before parathyroidectomy and at the sixth postoperative month.

Results

The difference in the NLR, serum parathormone, serum corrected calcium, and serum phosphate values ​​before and after parathyroidectomy was statistically significant (p=0.019, p<0.001, p<0.001, and p<0.001, respectively), but there was no significant difference in the WBC count ​​(p=0.314). The correlation analysis performed before parathyroidectomy revealed a significant positive correlation between NLR and serum parathormone (r=0.519, p=0.001), serum corrected calcium (r=0.390, p=0.017) and WBC count (r=0.531, p=0.001), and a significant negative correlation between NLR and serum phosphate (r=-0.331).

Conclusion

In patients with PHPT, successful parathyroidectomy results in a decrease in NLR. Increased systemic inflammation in patients with PHPT can be reduced following parathyroidectomy.

## Introduction

Primary hyperparathyroidism (PHPT) is an endocrine disease characterized by the excessive autonomic production of parathormone (PTH), increased serum PTH and calcium (Ca) levels as a result of the impaired regulation of Ca metabolism, or an inappropriate increase of Ca and PTH [1-3]. PTH plays an essential role mainly in the control of serum Ca homeostasis. PHPT has effects on inflammation and the cardiovascular system, and broad direct or indirect metabolic activities [4, 5].

Studies have found that there is a relationship between a high serum PTH level and various inflammatory markers [C-reactive protein (CRP), red cell distribution width (RDW), and platelet/lymphocyte ratio] [6]. Considering that inflammation is involved in the pathophysiology of cardiovascular disease, cancer, ageing, and many diseases, a relationship between PTH and inflammation is expected.

Studies show a complex interaction between inflammation and changes in PTH levels. High PTH levels have been shown in septic patients [7]. PTH promotes the production of interleukin-6 (IL-6) by stimulating osteoblasts and hepatocytes [8, 9], which, in turn, induces the synthesis of acute-phase reactants in the liver [3, 10]. Therefore, inflammation markers are expected to be high in patients with PHPT that present with high PTH.

Various biochemical and hematological markers are available to assess systemic inflammation, but some are costly to use. It is important that the marker to be used as an indicator of inflammation is inexpensive and easily available [11]. The neutrophil/lymphocyte ratio (NLR) is an inexpensive marker that can be easily calculated by dividing the neutrophil count by the lymphocyte count due to a simple blood test of systemic inflammation [12, 13]. However, conflicting results have been reported as increased, unchanged, or decreased inflammatory markers after parathyroidectomy (PTX) in patients with hyperparathyroidism [12, 14-18].

This study aimed to examine the pre- and post-PTX values ​​of NLR, which is an indicator of systemic inflammation in patients with PHPT, and compare its relationship with biochemical variables.

## Materials and methods

This research has received approval from the Adiyaman University Clinical Research Ethics Committee (protocol number: 2020/6-26, date: 23/06/2020) and included 37 patients who underwent successful PTX due to PHPT between June 2017 and June 2020 in the general surgery clinic of Adiyaman University Training and Research Hospital. The data of these patients were retrospectively analysed. Age, gender, serum Ca, serum PTH, serum albumin, serum phosphorus, white blood cell (WBC) count, neutrophil, and lymphocyte values were noted before PTX and the sixth postoperative month. The formula [(4-Albumin (gr/dl)) * 0.8 + serum Ca (mg/dl)] was used to calculate the corrected Ca value. NLR was obtained by simply dividing the neutrophil count by the lymphocyte count.

According to the 4th International Workshop guidelines, a multidisciplinary council made the decision to recommend PTX in patients with serum Ca level being 1 mg/dl higher than the upper limit of the normal range, those with decreased bone mineral density (a significant decrease in bone mineral density in lumbar vertebrae, hip bones, or distal radius, peak bone mass > 2.5 SD, and T score < -2.5), those with renal failure (glomerular filtration rate < 60 ml/min), nephrolithiasis cases, patients aged under 50 years, and those that did not want to or could not undergo long-term medical treatment [15].

Hypercalcemia that persisted after PTX or recurred within six months was accepted as persistent hyperparathyroidism, and cases that developed hypercalcemia after six months or later were considered as recurrent hyperparathyroidism. Patients with persistent and recurrent hyperparathyroidism, malignancy, diabetes mellitus, hyperthyroidism, multiple endocrine neoplasia, and systemic infections were excluded from the study.

Statistical analysis was carried out using the Statistical Package for the Social Sciences (version 25.0, IBM Corp., Armonk, NY, USA). Numerical variables were stated as the mean and standard deviation of minimum and maximum values, while categorical variables were obtained as numbers and percentages. The distribution of data was evaluated using the Shapiro-Wilk test. Pre- and post-PTX variables were analysed using the paired Student's t-test. Pearson’s correlation analysis was used to determine possible relationships between the variables. A p-value of less than 0.05 was considered statistically significant.

## Results

This retrospective study included 37 patients with PHPT who underwent successful PTX. Thirty-three (89.2%) of the patients were female, and four (10.8%) were male. The median age was 54 (min: 28, max: 84) years. There was a weak inverse correlation between the age of the patients and their preoperative serum PTH, serum phosphate, WBC count and NLR values (r = -0.179, r = -0.184, r = -0.092, and r = -0.164, respectively), but it was not at a statistically significant level. There was a weak correlation between the patients' age and the serum corrected Ca values ​​(r = 0.243), and this was also not statistically significant.

**Figure 1:**
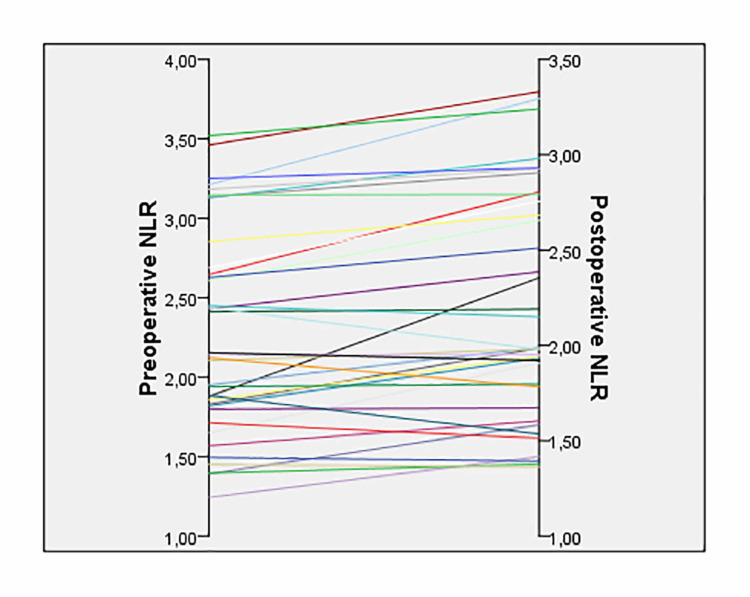
Preoperative and postoperative NLR values of the patients (p = 0.019) NLR: neutrophil/lymphocyte ratio

The mean preoperative serum corrected Ca value ​​of the patients was 13.69 ± 0.65 mg/dl (min: 10.8, max: 16.6), and the mean postoperative corrected mean Ca value ​​was 9.19 ± 0.52 mg/dl (min: 8.18-max: 9.9). There was a statistically significant difference between the preoperative and postoperative serum corrected Ca values ​​(p < 0.001). The mean preoperative and postoperative serum PTH values ​​of the patients were 214.61 ± 151.87 pg/ml (min: 74.1-max: 843.0) and 47.35 ± 17.30 pg/ml (min: 24.7-max: 98.6), respectively, indicating a statistically significant difference ​​(p < 0.001). The mean serum phosphate values of the patients were 2.39 ± 0.44 mg/dl (min: 1.60-max: 3.20) preoperatively and 3.30 ± 0.38 mg/dl (min: 2.50-max: 4.20) postoperatively, with a statistically significant difference ​​(p < 0.001). The mean preoperative WBC count ​​was 7.25 ± 1.03 (*103/µl) (min: 5.21-max: 8.93) while the mean postoperative WBC count ​​was 7.50 ± 1.18 (*103/µl) (min: 5.30-max: 9.27), revealing no statistically significant difference ​​(p = 0.314). The mean preoperative and postoperative NLR values ​​of the patients were 2.28 ± 0.65 (min: 1.24-max: 3.52) and 2.20 ± 0.59 (min: 1.24-max: 3.52), respectively. There was a statistically significant difference between the preoperative and postoperative NLR values ​​(p = 0.019) (Table 1) (Figure 1).

**Table 1:** Comparison of the laboratory variables of the patients before and after parathyroidectomy NLR: neutrophil/lymphocyte ratio

	Preoperative	Postoperative	P-value
Serum corrected calcium (mg/dl)	13.69 ± 1.67	9.19 ± 0.52	<0.001
Serum parathormone (pg/ml)	214.61 ± 151.87	47.35 ± 17.3	<0.001
Serum phosphate (mg/dl)	2.39 ± 0.44	3.30 ± 0.38	<0.001
White blood cell count (×103/µL)	7.25 ± 1.03	7.50 ± 1.18	0.314
NLR	2.28 ± 0.65	2.20 ± 0.59	0.019

Pearson’s correlation analysis was performed to evaluate the relationship between the preoperative serum PTH, serum corrected Ca, serum phosphate, WBC count, and NLR (Table 2).

**Table 2:** Pearson’s correlation analysis results of the relationship between variables before parathyroidectomy in patients with PHPT Pearson’s correlation *p < 0.05, **p < 0.01, ***p = 0.001; PTH: parathormone; WBC: white blood cell; Ca: calcium; NLR: neutrophil/lymphocyte ratio; PHPT: primary hyperparathyroidism

	Serum PTH	Serum corrected Ca	Serum phosphate	WBC count	NLR
Age	-0.179	0.243	-0.184	-0.092	-0.164
Serum PTH		0.286	-0.353^*^	0.231	0.539***
Serum corrected Ca			-0.435^**^	0.241	0.390^*^
Serum phosphate				-0.307	-0.331^*^
WBC count					0.531^***^

**Figure 2:**
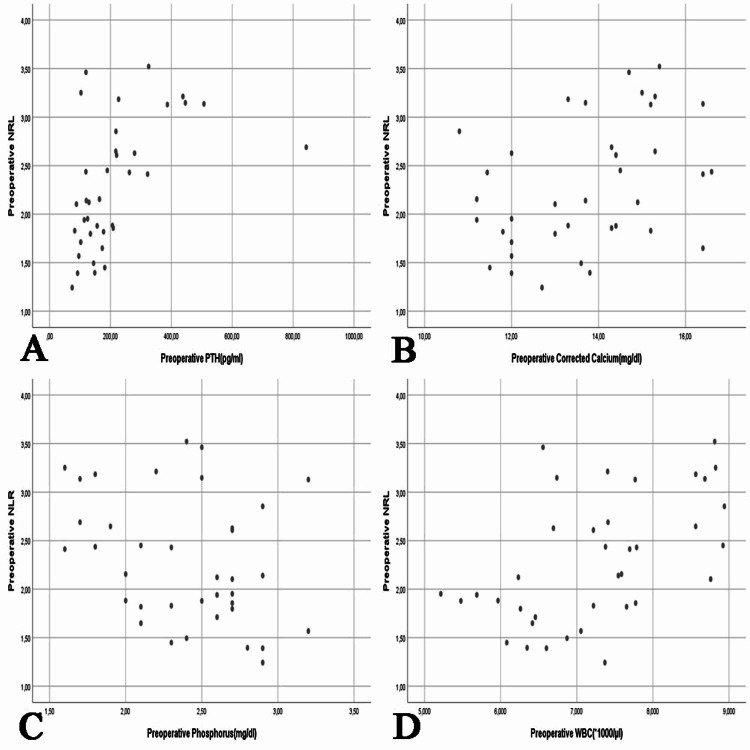
Comparison of the patients’ preoperative NLR with laboratory parameters. A: Preoperative PTH (p = 0.001), B: Preoperative corrected Ca (p = 0.017), C: Preoperative phosphate (p = 0.046), D: Preoperative WBC count (p = 0.001). PTH: parathormone; WBC: white blood cells; Ca: calcium; NLR: neutrophil/lymphocyte ratio

According to the results, the preoperative serum PTH had a weak non-significant correlation with serum corrected Ca ​​(r = 0.286), a weak non-significant inverse correlation with serum phosphate ​​(r = -0.353, p = 0.032), a weak non-significant correlation with the WBC count (r = 0.231), and a moderate significant positive correlation with NLR (r = 0.519, p = 0.001). The preoperative serum corrected Ca had a weak significant inverse correlation with serum phosphate (r = -0.435, p = 0.007), a weak non-significant correlation with the WBC count (r = 0.241), and a weak significant correlation with NLR (r = 0.390, p = 0.017). The preoperative serum phosphate had a non-significant inverse correlation with the WBC count ​​(r = -0.307) and a weak significant inverse correlation with NLR ​​(r = -0.331, p = 0.046). Lastly, there was a moderate significant correlation between the preoperative WBC count ​​and NLR ​​(r = 0.531, p = 0.001) (Figure 2).

## Discussion

Neutrophil and lymphocyte parameters are important inflammation indicators of the pathogenesis of many diseases presenting with systemic inflammation. In many studies, NLR has been proposed as a simple and readily available biomarker for inflammatory conditions, including systemic diseases such as malignancies, cardiovascular disorders, vertigo, diabetes mellitus, neurological disorders, rheumatological disorders, hypertension, and renal failure [11, 19-21]. Alemzadeh et al. stated that the serum PTH level was an independent determinant of chronic inflammation [16]. Stamatelopoulos et al. suggested that PHPT could increase blood pressure through PTH and inflammatory mediated mechanisms [22]. Yang et al. showed a decrease in NLR after successful PTX in patients with secondary hyperparathyroidism. They noted the regulatory effects of this procedure on systemic inflammation, which could also explain the beneficial role of PTX in anaemia, arterial stiffness, and survival in secondary hyperparathyroidism [23]. Lam et al. detected a relationship between PHPT and systemic inflammation and found that systemic inflammation was reversible after rapid treatment [18]. Similarly, in our study, evaluating patients with PHPT, in the sixth month after successful PTX, there was a significant decrease was observed in NLR, which is an indicator of systemic inflammation. Zeren et al. determined a positive correlation between preoperative PTH and NLR in patients with PHPT [11]. In our correlation analysis, we found a non-significant negative correlation between pre-PTX NLR and age and serum phosphate. We also observed a significant positive correlation between the NLR value and the serum PTH, serum corrected Ca, and WBC count variables.

Conflicting reports have been published in various studies attempting to explain the relationship between PTH and inflammatory markers. Emam et al. evaluated CRP and IL-6 as inflammatory biomarkers with high sensitivity in patients with asymptomatic PHPT. They reported that these values were higher in patients with asymptomatic PHPT than in the control group. Besides, the authors argued that serum PTH was significantly associated with inflammatory biomarkers, suggesting a subclinical inflammatory response in this patient group [24]. In a study investigating inflammatory biomarkers in patients with PHPT, Chertok-Shacham et al. reported found no significant correlation between the PTH or Ca levels and fibrinogen, D-dimer, CRP, and leukocyte [25]. ​​Our correlation analysis revealed a positive correlation between the serum PTH and serum corrected Ca and WBC values ​​before PTX, but this correlation was not significant.

The leukocyte count, which can be rapidly measured with a hemogram test, is frequently used in daily practice and is a well-known inflammation indicator [26]. Although Yang et al. found an increase in the WBC count after successful PTX in patients with hyperparathyroidism, this was not at a statistically significant level. In the same study, a decrease was observed in the WBC count after PTX in patients with persistent hyperparathyroidism, but this decrease was also non-significant [23]. Similarly, in our study, there was some increase in the WBC count after successful PTX, and it was not statistically significant (p = 0.316). However, a significant positive correlation was observed between the pre-PTX WBC count and NLR (r = 0.531, p = 0.001).

Toraman et al. noted a positive correlation between the NLR and serum phosphate values in patients with hyperparathyroidism, but this did not reach a level of statistical significance (r = 0.054, p = 0.347) [27]. Lam et al. found a negative correlation between the serum phosphate and NLR values before PTX in patients with PHPT. Still, they also noted that this was not at a statistically significant level [18]. In our study, we detected a significant negative correlation between the serum phosphate and NLR values ​​before PTX (r = 0.331, p = 0.046). We consider that this negative correlation was due to the low serum phosphate values ​​before surgery in patients with PHPT.

As expected, in patients with PHPT, the serum PTH and corrected Ca levels are higher, and the serum phosphate values are lower than the standard ranges [3]. In a study evaluating patients with hyperparathyroidism, Toraman et al. found a significant negative correlation between the NLR and serum Ca values (r = -0.146, p = 0.011) [27]. Lam et al. reported a positive and statistically significant correlation between the NLR and serum Ca values before PTX in patients with PHPT (r = 0.376, p < 0.01) [18]. In the current study, there was a statistically significant positive correlation between the pre-PTX serum corrected Ca and NLR values (r = 0.390, p = 0.017). We consider that this positive correlation was due to the high serum corrected Ca values ​​before PTX in patients with PHPT.

Our study's most important limitations concern its retrospective nature, small sample size, and the absence of a control group. Prospective studies to be conducted with larger patient populations may provide more meaningful results.

## Conclusions

Our study observed that NRL, a systemic inflammation marker, decreased after PTX in patients with PHPT. The data obtained from the study show that PTX reduces the risks of increased systemic inflammation, as well as providing treatment for patients with PHPT.
